# Addition of the Aldose Reductase Inhibitor Benzofuroxane Derivative BF-5m to Prolonged and Moderate Exercise Training Enhanced Protection of the Rat Heart From Type-1 Diabetes

**DOI:** 10.3389/fphar.2019.00392

**Published:** 2019-04-16

**Authors:** Bartolo Ferraro, Maria Donniacuo, Loredana Sodano, Franca Ferraraccio, Rosa Maisto, Eliana Gulotta, Gorizio Pieretti, Michele D’Amico, Maria Consiglia Trotta, Barbara Rinaldi

**Affiliations:** ^1^Institute for Cardiovascular Prevention (IPEK), Ludwig Maximilian University of Munich, Munich, Germany; ^2^DZHK, Partner Site Munich Heart Alliance, Munich, Germany; ^3^Department of Experimental Medicine, Pharmacology Division, University of Campania “L. Vanvitelli”, Naples, Italy; ^4^Department of Clinical, Public and Preventive Medicine, University of Campania “L. Vanvitelli”, Naples, Italy; ^5^Department of Surgical, Oncological and Stomatological Disciplines, University of Palermo, Palermo, Italy; ^6^Multidisciplinary Department of Surgical and Dental Specialities, University of Campania “L. Vanvitelli”, Naples, Italy

**Keywords:** diabetes, aldose-reductase, benzofuroxane, exercise, QT interval

## Abstract

Moderate exercise training may not be sufficient to exert beneficial effects on the cardiovascular system because of the long-term multifactorial etiology of diabetic complications. The addition of a proper pharmacological tool to the physical exercise should improve the outcomes of the diabetic damage. Here it is shown that 8 weeks exercise training of type 1 diabetic Sprague-Dawley (SD) rats resulted in a significantly increased heart rate, a 14% increase in the left ventricular ejection fraction (LVEF) increased plasma insulin levels and a 13% decrease in plasma glucose with respect to sedentary animals. The training also resulted in a 22% reduction in cardiac QT interval from a diabetic sedentary value of 185 ± 19 ms. Treatment of trained rats with the new antioxidant and NO-releasing aldose reductase 2 inhibitor 5(6)-(benzo[*d*]thiazol-2-ylmethoxy) benzofuroxane BF-5m, 20 mg/kg/day, added a further and significant (*P* < 0.01 vs. sedentary) increase of the LVEF up to 38% at 8 week time point. The long QT interval recorded in trained rats was reduced to further 12% by addition to the training of pharmacological treatment with 20 mg/kg/day BF-5m. At this time, the association of the two treatments improved the expression into the cardiac tissue of sarcoplasmic reticulum Ca^2+^ ATPase 2 (SERCA2) and manganese superoxide dismutase (MnSOD), and reduced the fibrosis.

## Introduction

Diabetes mellitus, a common disease in most developed countries, causes biochemical and pathological complications. Physical exercise training is recognized as a non-pharmacological tool to prevent the pathological cardiac consequences of diabetes (Verboven et al., 2018; Yousefi and TaheriChadorneshin, 2018), including blood pressure control, cardiac remodeling after myocardial infarction, heart failure, reduced cardiac contractility (Wisloff et al., 2002; Almeida et al., 2014; Liu et al., 2018), insulin resistance, dyslipidaemia, increased pro-inflammatory cytokines (Abd El-Kader et al., 2013), reduction of the oxidative response, and reduced antioxidant activity within the myocardium (Barbosa et al., 2012; Kraljevic et al., 2013; Bei et al., 2017). However, exercise training may not be sufficient to exert beneficial effects on the cardiovascular system because of the long-term multifactorial etiology of diabetic complications. Previous studies have shown that hyperglycaemia causes an imbalance between free radicals and antioxidants, resulting in oxidative stress (Yaribeygi et al., 2018). Addition of a proper pharmacological agent to physical exercise should improve outcomes related to diabetic damage. In this context, it is investigated here the addition of the selective aldose reductase 2 inhibitor benzofuroxane derivative, BF-5m, (Sartini et al., 2012) to prolonged moderate exercise training. BF-5m effectively treats diabetic symptoms *in vitro* (Sartini et al., 2012) and in models of inflammation-based pathologies (Di Filippo et al., 2014), since aldose reductase 2 is the rate-limiting enzyme of the polyol pathway in diabetes. Furthermore, BF-5m may facilitate control of diabetes complications.

## Materials and Methods

### Drug

BF-5m, 5(6)-(benzo[*d*]thiazol-2-ylmethoxy) benzofuroxane, was synthesized at the Department of Pharmacy of the University of Pisa, Italy, as reported previously (Sartini et al., 2012; Di Filippo et al., 2014). Briefly, alkylation of commercially available 4-amino-3-nitrophenol with chloroacetonitrile, in the presence of anhydrous potassium carbonate, resulted in 2-(4-amino-3-nitrophenoxy)acetonitrile, which was reacted with o-aminothiophenol to provide the key intermediate 4-[(benzo[*d*]thiazol-2-yl)methoxy]-2-nitrobenzenamine. Treatment with sodium nitrite in concentrated hydrochloric acid, and then with sodium azide in water, resulted in azido-derivative, which was cyclized to the target inhibitor, 5(6)-(benzo[*d*]thiazol-2-ylmethoxy) benzofuroxane, when heated under reflux in acetic acid.

### Animals

Male SD rats (180–220 g) purchased from Envigo (Italy) were housed in individual cages under controlled conditions (12–12 h light-dark cycle; room temperature 20–22°C; humidity 55–60%), fed a standard chow diet and free access to food and water. All experimental procedures were approved by the Animal Ethics Committee of University of Campania “L. Vanvitelli” of Naples. Animal care was in compliance with Italian (D.L. 116/92) and European Commission (O.J. of E.C. L358/1 18/12/86) guidelines on the use and protection of laboratory animals. All efforts were made to minimize animal suffering and to reduce the number of animals used.

### Induction of Type I Diabetes Mellitus

Sprague-Dawley rats were treated with a single intraperitoneal injection of STZ (65 mg/kg) (Sigma Chemical Co., St. Louis, MO, United States) dissolved in 0.1 M citrate buffer (pH 4.3) in order to reproduce type-1 diabetes (Furman, 2015). Development of diabetes was confirmed 7 days after injection of STZ by measurement of plasma glucose levels. Rats with plasma glucose levels of 200 mg/dl or higher were included in the study (Ozkol et al., 2013; Furman, 2015). This time point was considered time 0 (T0) and marked the beginning of the study.

### Experimental Design

**FIGURE 1:**
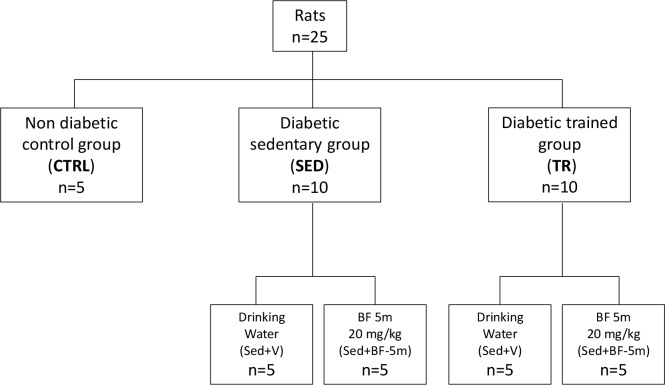
Flow chart showing the experimental design. CTRL, healthy control rats; Sed+V, diabetic sedentary rats treated with vehicle; Sed+BF-5m, diabetic sedentary rats treated with BF-5m 20 mg/kg/day; Tr+V, diabetic trained rats treated with vehicle; Tr+BF-5m, diabetic trained rats treated with BF-5m 20 mg/kg/day.

Twenty-five rats were divided into three main groups: non diabetic as healthy controls (*n* = 5), diabetic sedentary (*n* = 10) and diabetic exercise trained (*n* = 10). Sedentary and trained rats were randomly allocated into two subgroups: (1) five rats received drinking water only, and (2) five rats treated with 20 mg/kg/day of 5(6)-(benzo[*d*]thiazol-2-ylmethoxy) benzofuroxane (referred as BF-5m) dissolved in 1% DMSO in drinking water (Figure 1). This dose was chosen following in lab experience and resembling the dose of the most known ALR2 inhibitor sorbinil in STZ-rats (Körner et al., 1992; Mizuno et al., 1992; Ward et al., 2005). BF-5m was administered from T0 for 8 weeks.

The training protocol was executed as previously described (Rinaldi et al., 2013). Briefly, the trained group was acclimatized to training by walking 10 min/day at a speed of 15 m/min on a treadmill for 2 weeks (Panlab/Harvard Apparatus Treadmills, Holliston, MA, United States). After the acclimatization period (3rd week), running was gradually increased to 30 m/min for 45 min/day, 5 days/week, for additional 6 weeks. A horizontal shock grid that delivered 1 mA was placed 10 cm from the rear of the chamber to provide stimulus for the animals to run. The rats were placed on the grid treadmill cell and the exercise capacity of each rat was measured by the effective time of exercise calculated as the difference between the total running time of each rat and the time spent on the shock grid (Lightfoot et al., 2001). The sedentary rats remained in cages for the entire duration of the training protocol. All drug treatments were started on the first day of experiments.

### Transthoracic Echocardiographic and ECG Measurements

Before, during (week 5 of the study), and at the end of the study (week 8 of the study), cardiac function was evaluated in anesthetized rats using non-invasive transthoracic M-Mode echocardiography (Visual SONICS VeVo 770 imaging system with a RMV710B; Toronto, Ontario, Canada). Rats were anesthetized for about 30–45 min with an intramuscular injection of ketamine hydrochloride (100 mg/kg) and medetomidine (0.25 mg/kg). At 5 weeks, atipamezole was used as anesthetic reversal in the same medetomidine volume. Transthoracic echocardiographic determinations (LVEF%; LVFS% and HR bpm) were performed in the lateral decubitus position as previously reported (Rinaldi et al., 2013). A 3-min ECG (speed 50 mm/s) was recorded at 5 and 8 week time points. After the completion of all the training and echocardiographic procedures the rats, still under anesthesia, had the chest rapidly and carefully opened longitudinally at the level of the sternum, avoiding the mammary artery. The hearts were then excised and posed on dry ice.

### Western Blot Analyses

Western blot analyses were performed on the heart left ventricle free wall muscle to assess SERCA2, and MnSOD expression. Heart samples were homogenized on ice using RIPA buffer (Santa Cruz Biotechnology, Milan, Italy) including a protease inhibitor cocktail (Roche Diagnostics, Mannheim, Germany). Homogenates were centrifuged at 12,000 x g and the supernatants were collected. Protein concentration was determined using the Bio-Rad protein assay (Bio-Rad Laboratories, Milan, Italy). A total of 100 μg of protein per sample was separated on denaturing 8% SDS-PAGE gels and transferred to PVDF membranes. Membranes were blocked for 1 h at room temperature with 5% milk in T-TBS (tris buffered saline with 0.1% Tween 20), followed by incubation at 4°C overnight with primary antibodies against SERCA2 (1:1000, 3625 Abcam, Cambridge, United Kingdom), and MnSOD (1:1000, 06-984 Millipore, United States). Membranes were then washed three times with 0.1% T-TBS solution, and incubated for 1 h at room temperature with a secondary antibody donkey polyclonal anti-rabbit IgG-HRP (1:5000, sc-2004 Santa Cruz Biotechnology, Milan, Italy) or goat anti-mouse IgG-HRP (1:5000, sc-2005 Santa Cruz Biotechnology, Milan, Italy), according to the primary antibodies data sheet (Santa Cruz Biotechnology, Milan, Italy). GAPDH antibody (1:5000, G8795 Sigma-Aldrich, Milan, Italy) was used as an internal standard. Immunoreactive bands were visualized using an enhanced chemiluminescence system (SuperSignal West Femto Maximum Sensitivity Substrate, Pierce, Rockford, IL, United States). Protein bands were scanned and quantified with Gel Doc-2000 (Bio-Rad, Milan, Italy).

### ELISA

To assess the levels of circulating insulin in the blood we used the Ultrasensitive Rat Insulin ELISA kit (Mercodia AB, Uppsala, Sweden). Briefly, blood was collected from the tail vein and centrifuged at 3000 ×*g* for 10 min to obtain serum which was immediately processed, according to the manufacturer’s protocol.

### Immunohistochemistry

Paraffin-embedded sections were used according to previously published methods (Rossi et al., 2012). Sections were incubated with a specific antibody anti-vimentin (1:1000, 137321 Abcam, Cambridge, United Kingdom). Sections were then washed with PBS and incubated with secondary antibodies. Specific labeling was detected with a biotin-conjugated goat anti-rabbit IgG and avidin-biotin peroxidase complex (1:200, BA-1000 DBA, Milan, Italy). Five distinct preparations were analyzed by a pathologist who was familiar with the protocol. A total of 23 fields of view were analyzed in each section for a total area of 4.3526e + 0.04 μm^2^ at 400× magnification. Using computer-aided planimetry (IM500, Leica Microsystem, Milan, Italy) the percentage of positive stained area per total area analyzed was calculated. A colored threshold mask for immunostaining was defined and applied to all sections.

In addition, cardiac tissue was stained with haematoxylin and eosin and observed at a magnification of 200× and 400× using an optical microscope to evaluate morphology.

### Statistical Analysis

The data obtained were analyzed by ANOVA for normally distributed data, and Kruskal-Wallis test for non-normally distributed data. Bonferroni test was used to make pairwise comparisons. *P* < 0.05 was considered significant and SPSS2 software used.

## Results

### Effects of Prolonged Moderate Exercise Training + Pharmacological BF-5m Treatment on Physical, Echocardiographic and Haemodynamic Parameters

**FIGURE 2:**
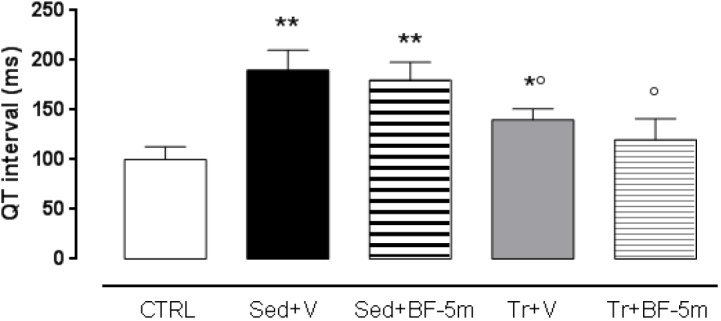
Eight weeks cardiac QT interval in non-diabetic rats, sedentary diabetic rats or in response to a prolonged moderate exercise training. Results are reported as mean of five observations ± S.E.M. CTRL, healthy control rats; Sed+V, diabetic sedentary rats treated with vehicle; Sed+BF-5m, diabetic sedentary rats treated with BF-5m 20 mg/kg/day; Tr+V, diabetic trained rats treated with vehicle; Tr+BF-5m, diabetic trained rats treated with BF-5m 20 mg/kg/day; ^∗^*P* < 0.05 and ^∗∗^*P* < 0.01 vs. CTRL; °*P* < 0.05 vs. the sedentary group with equivalent pharmacological treatment.

Table 1 shows that there were no significant differences between the physical parameters of trained diabetic rats and sedentary diabetic rats. However, the trained group showed significantly (*P* < 0.05) increased HR and the LVEF compared to the sedentary group. 8 weeks treatment of sedentary rats with BF-5m significantly increased LVEF with respect to the untreated sedentary rats. In trained rats, the addition of BF-5m increased LVEF by up to 38% compared to the sedentary vehicle-treated rats, and up to 16% compared to the trained-vehicle treated rats. As expected, high blood glucose levels led to a prolonged QT interval (e.g., 185 ± 19 ms) in sedentary rats. At the same time point this effect was decreased by up to 22% as a result of a moderate exercise training, and was decreased by up to 34% by the addition of 20 mg/kg/day BF-5m to exercise training (Figure 2).

**Table 1:** Physical and cardiac parameters of diabetic rats after 5 and 8 weeks observation.

	CTRL	5 week diabetic			
		Sed+V	Sed+BF-5m	Tr+V	Tr+BF-5m
**Physical data**					
Body wt (g)	250 26	219 27	227 30	220 24	218 14
Heart wt (g)	0.43 0.04	0.53 0.09	0.6 0.05*	0.62 0.03**	0.61 0.02**
Heart wt.body wt^-1^ (g. kg^-1^)	1.72 0.003	2.42 0.002**	2.64 0.002**	2.58 0.004**	2.53 0.003**
Blood glucose (mg.dl^-1^)	86 19	422 20**	412 35**	394 12**	385 10**
**Hemodynamic and LV data**					
Heart rate (beats/min)	360 15	194 9**	209 28**	254 30^∗∗∘^	240 18^∗∗∘^
LVFS %	64 3.3	45 2.1**	49 4.3*	52 2^∗∘^	52 3^∗∘^
LVEF %	84 5	62 4**	68 2.7*	67 2.1*	72 3^∗∘^

		**8 week diabetic**			
**Physical data**					
Body wt (g)	280 15	222 29*	247 12*	221 37	219 39
Heart wt (g)	0.46 0.03	0.57 0.1	0.6 0.04*	0.62 0.06*	0.6 0.1
Heart wt.body wt^-1^ (g. kg^-1^)	1.64 0.03	2.57 0.02**	2.42 0.001**	2.8 0.001**	2.74 0.002**
Blood glucose (mg.dl^-1^)	85 18	462 35**	438 34**	403 15**	406 34**
**Hemodynamic and LV data**					
Heart rate (beats/min)	358 12	187 21**	236 35**	303 27^∗∘∘^	246 17^∗∗∘^
LVFS %	65 2.8	41 2.3**	47 1.4^∘∗∗^	49 1.2^∘∘∗∗^	50 2^∗∗∘∘^
LVEF %	85 4	58 3.4^∗∗∘^	66 1.2^∗∗∘^	69 1.8^∘∗∗^	80 1^∘∘§^


*Sed, Sedentary; V, vehicle; BF-5m, 5(6)-(benzo[*d*]thiazol-2-ylmethoxy) benzofuroxane; Tr, trained; LV, left ventricle; LVFS, Left ventricular fraction shortening; LVEF, Left ventricular ejection fraction. Results are reported as mean of five observations ± S.E.M. CTRL, healthy non-diabetic control rats; Sed+V, diabetic sedentary rats treated with vehicle; Sed+BF-5m, diabetic sedentary rats treated with BF-5m 20 mg/kg/day; Tr+V, diabetic trained rats treated with vehicle; Tr+BF-5m, diabetic trained rats treated with BF-5m 20 mg/kg/day. ^∗^*P* < 0.05 and ^∗∗^*P* < 0.01 vs. CTRL; °*P* < 0.05 vs. Sed+V; ^∘∘^*P* < 0.01 vs. Sed+V; §*P* < 0.05 vs. values recorded at 5 weeks.*

### Effects of Exercise Training and BF-5m Treatment on Circulating Insulin and Plasma Glucose Levels

**FIGURE 3:**
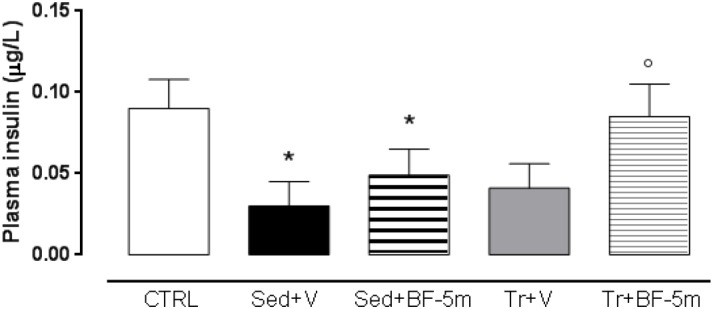
Eight weeks plasma insulin levels in healthy control rats, sedentary diabetic rats or those subjected to a prolonged moderate exercise training. Results are reported as mean of five observations ± S.E.M. CTRL, healthy control rats; Sed+V, diabetic sedentary rats treated with vehicle; Sed+BF-5m, diabetic sedentary rats treated with BF-5m 20 mg/kg/day; Tr+V, diabetic trained rats treated with vehicle; Tr+BF-5m, diabetic trained rats treated with BF-5m 20 mg/kg/day; ^∗^*P* < 0.05 vs. CTRL; °*P* < 0.05 vs. the same pharmacological treatment in the sedentary group.

Plasma ELISA showed that sedentary diabetic rats had lower levels of insulin across the study time points. Insulin levels were not significantly affected by either exercise training or by BF-5m alone (Figure 3). Interestingly, treatment with BF-5m combined with physical activity significantly increased (e.g., 133%, *P* < 0.05) plasma insulin levels at the 8-week time point (Figure 3). Consequently, plasma glucose levels were significantly (*P* < 0.05) decreased by 12% (Table 1).

### Effects of Moderate Exercise Training and BF-5m Treatment on SERCA2 and MnSOD Expression

**FIGURE 4:**
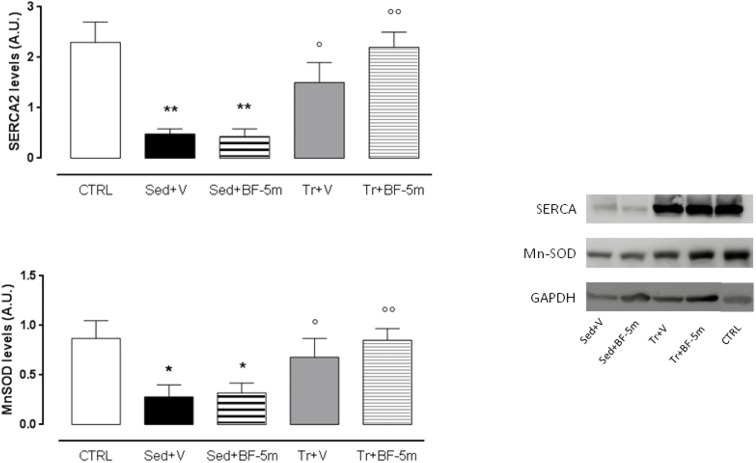
Western blot of SERCA2 and MnSOD. Experimental treatment were performed as described in Figure 1. Results are reported as mean of five observations ± S.E.M. CTRL, healthy control rats; Sed+V, diabetic sedentary rats treated with vehicle; Sed+BF-5m, diabetic sedentary rats treated with BF-5m 20 mg/kg/day; Tr+V, diabetic trained rats treated with vehicle; Tr+BF-5m, diabetic trained rats treated with BF-5m 20 mg/kg/day; A.U., arbitrary units; ^∗^*P* < 0.05 and ^∗∗^*P* < 0.01 vs. CTRL; °*P* < 0.05 and ^∘∘^*P* < 0.01 vs. the same pharmacological treatment in the sedentary group.

Western blot analysis (Figure 4) showed that SERCA2 and MnSOD expression were significantly higher in the trained diabetic rats compared to the sedentary diabetic rats (*P* < 0.05). SERCA2 and MnSOD were elevated 4-fold and 2-fold, respectively, in rats subject to the combined treatment of prolonged exercise training and 20 mg/kg/day BF-5m as compared to the sedentary rats (*P* < 0.01 vs. sedentary rats).

### Effects of BF-5m Treatment on Cardiac Morphology and Development of Cardiac Fibrosis

**FIGURE 5:**
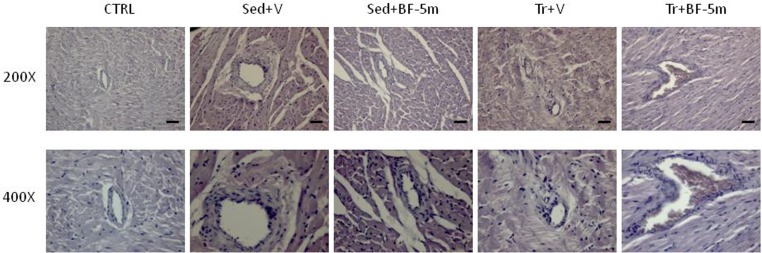
Representative hematoxylin and eosin staining of hearts from healthy control, diabetic sedentary and diabetic trained rats treated with vehicle, BF-5m or vehicle/BF-5m in combination with prolonged (8 weeks total) moderate exercise training (*N* = 5 preparations per group). CTRL, healthy control rats; Sed+V, diabetic sedentary rats treated with vehicle; Sed+BF-5m, diabetic sedentary rats treated with BF-5m 20 mg/kg/day; Tr+V, diabetic trained rats treated with vehicle; Tr+BF-5m, diabetic trained rats treated with BF-5m 20 mg/kg/day; Scale bar = 100 μm.

**FIGURE 6:**
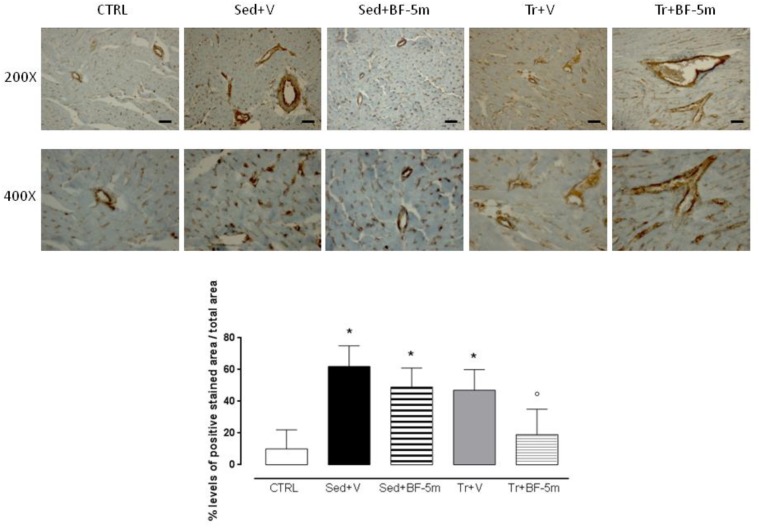
Heart pericapillary fibrosis and interstitial mesenchymal cells after 8 weeks of diabetes following staining with anti-vimentin antibody have been observed increased in the sedentary groups and in untreated trained rats. Prolonged moderate exercise training in association with BF-5m treatment causes a significant reduction in the percentage of fibrosis as shown by vimentin staining at 200X and 400X magnification. Results are reported as mean of five preparations ± S.E.M. CTRL, healthy control rats; Sed+V, diabetic sedentary rats treated with vehicle; Sed+BF-5m, diabetic sedentary rats treated with BF-5m 20 mg/kg; Tr+V, diabetic trained rats treated with vehicle; Tr+BF-5m, diabetic trained rats treated with BF-5m 20 mg/kg/day; Scale bar = 100 μm; ^∗^*P* < 0.05 vs. CTRL; °*P* < 0.05 vs. Sed+V.

Haematoxylin ad eosin staining performed on heart rat samples after 8 weeks of diabetes showed clear evidence of a structural damage with few signs of normal tissue organization. In contrast, minimal structural damage was observed in trained rat hearts and nearly the entire structure of the cardiac tissue in the combined exercise and BF-5m group was normal (Figure 5). Pericapillary fibrosis and increased interstitial mesenchymal cells were observed following staining with anti-vimentin antibody in the sedentary group. We showed that in trained rats there was a small amount of perivascular fibrosis and some fibroblasts and fibrocytes were present in the interstitial space. In addition, BF-5m treatment reduced fibrosis, with remained fibrosis localized to pericapillary and perivascular areas (Figure 6). Interestingly, exercise training and BF-5m resulted in a marked reduction of perivascular and interstitial fibrosis, accompanied by decreased number of interstitial fibrocytes (Figure 6).

## Discussion

The present study showed that the benzofuroxane derivative molecule BF-5m, combined with prolonged moderate physical exercise enhances heart protection from type-1 diabetes.

Physical exercise, especially aerobic exercise, is an integral part of diabetes treatment plans, because it improves glycaemic control, helps to maintain optimal body weight, and reduces the risk of cardiovascular disease (Laughlin et al., 2012; Improta Caria et al., 2018). Physical activity is an universally accepted treatment strategy that is often used in combination with pharmacological approaches, but also as a monotherapy for treatment of metabolic disorders, and leads to reduction of oxidative stress, (Leal et al., 2018) which is a contributor to development, and subsequent complications of diabetes (Le Lay et al., 2014; Shaw et al., 2014). In this context, the effectiveness of BF-5m, a benzofuroxane derivative, which inhibits aldose reductase (ALR2), was investigated here. ALR2, along with the sorbitol dehydrogenase, is part of the polyol pathway in which aldose reductase converts glucose to sorbitol and sorbitol dehydrogenase converts sorbitol into fructose, using NADPH and NAD^+^ as cofactors. ALR2 is an ubiquitous cytosolic protein comprised of 315 amino acid residues, and is the rate limiting enzyme in the polyol pathway (Maccari and Ottanà, 2014). ALR2 is active in hyperglycaemic conditions, when glycolytic capacity of the cell is saturated. ALR2 has low affinity for glucose, which must be present in excess in the cytoplasm to cause ALR2 activation (Sartini et al., 2012). Although the physiological functions of ALR2 have not been fully characterized, the non-uniform distribution of this enzyme in different anatomic regions appears to support the hypothesis that this enzyme plays cell-type specific roles. In non-insulin-sensitive tissues and those that passively transport glucose into cells (nerve, lens, kidneys, blood vessels), persistent extracellular hyperglycaemia leads to increased intracellular glucose. This increase, leads to activation of the polyol pathway by ALR2 (Di Filippo et al., 2014), which can result in oxidative and metabolic imbalances and subsequent tissue damage. In the heart, brain, and kidneys excess sugar results in increased production of free radicals and increased oxidative stress, which can overwhelm innate antioxidant systems (Mandavia et al., 2012). Inhibition of ALR2 could be an effective therapeutic strategy for treatment of diseases induced by hyperglycaemia. Although many pharmacological agents have yielded poor results in clinical trials, discovery of effective drugs to inhibit the ALR2 pathway and improve diabetic complications is critical. Many derivatives of benzofuroxane inhibit ARL2 activity (Sartini et al., 2012), but some also function as NO donors and scavengers of free radicals. Among these, the derivative benzyloxy-5m seems the most promising because it inhibits ALR2, spontaneously generates NO, and scavenges hydroxyl radicals. In this study, treatment of diabetic rats with ALR2 inhibitor BF-5m combined with prolonged exercise provided superior cardioprotection, as evidenced by prevention of onset and progression of cardiovascular dysfunction induced by hyperglycaemia. Co-treatment with BF-5m and exercise was more effective than either treatment alone. An explanation of this phenomenon may reside in a synergic action of BF-5m with the physical exercise against the oxidative stress and ROS overproduction induced by diabetes. Functional to this point it is well known beneficial effect of moderate exercise training on the increased formation of advanced glycation end-products (AGEs) and on the activation of protein kinase C (PKC) (Korzick et al., 2004; Gu et al., 2014), other two important pathways involved in diabetic ROS overproduction. BF-5m combined with the moderate exercise training may overwhelm the oxidative imbalance in diabetic rats if compared to the inhibition of the aldose reductase 2 alone.

The advantages of the combined therapies were as follow: cardiac QT was normalized to a greater extent in response to co-treatment; cardiac ejection fraction was increased by 33% in response to co-treatment compared to sedentary vehicle-treated rats, with a good recovery of systolic function; levels of SERCA2 and MnSOD were significantly increased in response to co-treatment with BF-5m and exercise; and insulin release was increased in the co-treatment group. Furthermore, immunohistochemical analysis using anti-vimentin antibodies showed significant improvement in pericapillary and perivascular cardiac fibrosis, and a slight decrease in interstitial mesenchymal cells. Although this study was limited by the small animal number, the still hypothetical BF-5m molecular mechanism and the clinical incidence of this pathology is less than 10% compared to the majority of type 2 diabetes, BF-5M combined with physical activity enhances the protection of the rat heart in this experimental model of type 1 diabetes.

## Ethics Statement

All the experimental procedures were approved by the Animal Ethics Committee of University of Campania “L. Vanvitelli” of Naples. Animal care was in compliance with Italian (D.L. 116/92) and European Commission (O.J. of E.C. L358/1 18/12/86) guidelines on the use and protection of laboratory animals. All efforts were made to minimize animal suffering and to reduce the number of animals used.

## Author Contributions

BF, MD, and LS performed the experimental research. MT and RM performed the data analysis. EG and GP took heart samples. FF performed the immunohistochemistry experiments. MD’A and BR wrote and reviewed the manuscript.

## Conflict of Interest Statement

The authors declare that the research was conducted in the absence of any commercial or financial relationships that could be construed as a potential conflict of interest.

## Abbreviations

ALR2: aldose reductase
BF-5m: aldose reductase 2 inhibitor 5(6)-(benzo[*d*]thiazol-2-ylmethoxy) benzofuroxane
HR: heart rate
LVEF: left ventricular ejection fraction
LVFS: left ventricular fraction shortening
MnSOD: manganese superoxide dismutase
NADPH: nicotinamide adenine dinucleotide phosphate
SD: Sprague-Dawley
SERCA2: sarcoplasmic reticulum Ca2+ ATPase 2
STZ: streptozotocin
